# Assessment of posterior tongue mobility using lingual‐palatal suction: Progress towards a functional definition of ankyloglossia

**DOI:** 10.1111/joor.13144

**Published:** 2021-01-17

**Authors:** Soroush Zaghi, Shayan Shamtoob, Cynthia Peterson, Loree Christianson, Sanda Valcu‐Pinkerton, Zahra Peeran, Brigitte Fung, Daniel Kwok‐keung Ng, Triin Jagomagi, Nicole Archambault, Bridget O’Connor, Kathy Winslow, Miche’ Lano, Janine Murdock, Lenore Morrissey, Audrey Yoon

**Affiliations:** ^1^ The Breathe Institute Los Angeles CA USA; ^2^ Happy Kids Dental Planet Agoura Hills CA USA; ^3^ Kwong Wah Hospital Hong Kong Hong Kong; ^4^ Hong Kong Sanatorium & Hospital Hong Kong Hong Kong; ^5^ Institute of Dentistry and Unimed United Clinics University of Tartu Tartu Estonia; ^6^ Minds in Motion Santa Monica CA USA; ^7^ O’Connor Dental Health Cork Ireland; ^8^ Independent Researcher Half Moon Bay CA USA; ^9^ South County Pediatric Speech Mission Viejo CA USA; ^10^ Be Well Collaborative Care Huntington Beach CA USA; ^11^ Division of Growth and Development Section of Pediatric Dentistry UCLA School of Dentistry Los Angeles CA USA

**Keywords:** ankyloglossia, classification of ankyloglossia, frenulum, functional ankylglossia, grading scale, lingual‐palatal suction, myofunctional, myofunctional therapy, oro‐facial myofunctional disorder, oromyofascial dysfunction, posterior tongue mobility, tongue‐tie

## Abstract

**Background:**

A functional definition of ankyloglossia has been based on assessment of tongue mobility using the tongue range of motion ratio (TRMR) with the tongue tip extended towards the incisive papilla (TIP). Whereas this measurement has been helpful in assessing for variations in the mobility of the anterior one‐third of the tongue (tongue tip and apex), it may be insufficient to adequately assess the mobility of the posterior two‐thirds body of the tongue. A commonly used modification is to assess TRMR while the tongue is held in suction against the roof of the mouth in lingual‐palatal suction (LPS).

**Objective:**

This study aims to explore the utility and normative values of TRMR‐LPS as an adjunct to functional assessment of tongue mobility using TRMR‐TIP.

**Study Design:**

Cross‐sectional cohort study of 611 subjects (ages: 3‐83 years) from the general population.

**Methods:**

Measurements of tongue mobility using TRMR were performed with TIP and LPS functional movements. Objective TRMR measurements were compared with subjective self‐assessment of resting tongue position, ease or difficulty elevating the tongue tip to the palate, and ease or difficulty elevating the tongue body to the palate.

**Results:**

There was a statistically significant association between the objective measures of TRMR‐TIP and TRMR‐LPS and subjective reports of tongue mobility. LPS measurements were much more highly correlated with differences in elevating the posterior body of the tongue as compared to TIP measurements (R^2^ 0.31 vs 0.05, *P* < .0001).

**Conclusions:**

This study validates the TRMR‐LPS as a useful functional metric for assessment of posterior tongue mobility.

## 1 INTRODUCTION

Restricted tongue mobility has long been appreciated to impact speech,^1^, ^2^ feeding^3^, ^4^ and oral hygiene^5^ and more recently has also been potentially implicated in maxillofacial development,^6^, ^7^ mouth breathing,^8^ myofascial tension^9^ and even sleep‐disordered breathing.^10^ ^,^ ^11^Whereas ankyloglossia (tongue‐tie) has been described as a condition of restricted tongue mobility caused by a restrictive lingual frenulum,^12^ there are many other causes for impaired tongue mobility (such as airway obstruction and lack of generalised practice, as well as inadequate tongue space and extraoral fascial restrictions, among other factors) that are often underappreciated.^9^ The term ‘functional ankyloglossia’ is used to characterise limitations of tongue mobility that may or may not be directly attributable to a structural restriction in the lingual frenulum.^13^

The lingual frenulum is a dynamic three‐dimensional structure formed by a central fold in a layer of fascia that extends across the floor of the mouth with high degree of morphologic variability between different individuals.^14^ The presence or absence of a short or tight lingual frenulum alone may or may not be directly associated with impairments of tongue mobility.^15^ Many patients with restrictive lingual frenulum may have only minor difficulties and may compensate for limitations in tongue movement.^16^ Patients may compensate for tongue movement, for example by lifting the mandible and/or the floor of the mouth.^9^ The compensations, in some cases, may not be benign and can be the genesis of future oro‐facial myofunctional or temporomandibular disorders.^33^

The word ‘ankyloglossia’ (ie tongue‐tie) is etymologically derived from ancient Greek by the words ‘ankúlos’ which means ‘to bend’ or ‘crooked, curved, rounded’ and ‘*glôssa’,* which refers to the ‘*tongue’;* as such ankyloglossia most appropriately refers to alterations in the mobility of the tongue that may sometimes be attributable to a tight or short lingual frenulum. According to a recent clinical consensus statement among otolaryngologists on ankyloglossia,^12^ however, there appears to be a bias towards considering restrictions of the lingual frenulum as the primary or sole determinant of tongue mobility. One of the biggest limiting factors for clinical research on the topic of ‘functional ankyloglossia’ is the paucity of objective measurements to define the presence or absence of the condition. Most definitions of the condition are based on structural characterisations of the lingual frenulum^15^, ^17^, ^18^, ^19^ or subjective descriptions of mobility,^20^, ^21^, ^22^, ^23^, ^24^ as there are limited objective tools to actually quantify functional variations in tongue mobility on a continuous numeric scale.^25^

Recently, our group demonstrated the need for moving towards a functional definition of ankyloglossia based on assessment of tongue mobility.^9^, ^13^ The tongue range of motion ratio (TRMR) based on work by Irene Marchesan^25^ was validated as a useful tool for the assessment of tongue mobility in children, adolescents and adults.^13^ The tool is based on a ratio of vertical extension of the tongue to the incisive papilla (TIP) in comparison with the maximal interincisal mouth opening. Whereas this measurement has been helpful in serving as an initial screening tool to assess for variations in the mobility of the anterior one‐third of the tongue (tongue tip and apex), we hypothesise that the measurement may be insufficient to adequately assess the mobility of the posterior two‐thirds (or body) of the tongue. A commonly used modification is to assess the tongue range of motion while the tongue is held in suction against the roof of the mouth in lingual‐palatal suction (LPS). Tongue strength can also be assessed by measuring the endurance with which the patients are able to sustain this posture. This manuscript aims to explore the utility and normative values of LPS as an objective tool for assessing the mobility and endurance of the posterior two‐thirds body of the tongue.

## 2 METHODS

### 2.1 Study design

Cross‐sectional multicenter cohort study of subject ages three and up from the general population surveyed in a standardised fashion by interdisciplinary professionals trained in the evaluation of oro‐facial myofunctional disorders at 10 sites including researchers in the United States, Hong Kong, Estonia and Ireland as part of the Functional Airway Evaluation Screening Tool (FAIREST) study. The study was approved by Solutions IRB on 3‐16‐18; IRB Protocol # 2018/03/4. Data were collected between 22 March 2018 and 5 August 2018. Subjects recruited include friends, family, colleagues and private clients of the researchers who volunteered without financial compensation and provided written informed consent to participate. Exclusion criteria were syndromic craniofacial disorder (eg Downs, Treacher Collins, Crouzon, Apert); history of tracheostomy dependence; prior history of laryngeal, subglottic, or pulmonary airway stenosis or surgery; pregnant women; mentally/emotionally/developmentally disabled; impaired decision‐making capacity; and prisoners. There were 21 objective screening tool items and an 8‐item subjective screening tool questionnaire completed by both subject and a FAIREST researcher. (See Supplement for FAIREST‐21 Questionnaire; also available online at http://www.FAIREST.org).

### 2.2 Objective assessment of tongue mobility

Step 1: Measurement of maximum interincisal mouth opening with the mouth opened as wide as possible without pain or discomfort, that is comfortable mouth opening (CMO).

Step 2: Measurement of the maximum interincisal mouth opening while the tongue tip is extended to the incisive papilla (TIP).

Step 3: Measurement of the maximum interincisal mouth opening while the tongue body is held against the palate in lingual‐palatal suction (LPS).

Step 4: TRMR‐TIP is calculated as a percentage of TIP divided by CMO.

Step 5: TRMR‐LPS is calculated as a percentage of LPS divided by CMO.

**FIGURE 1:**
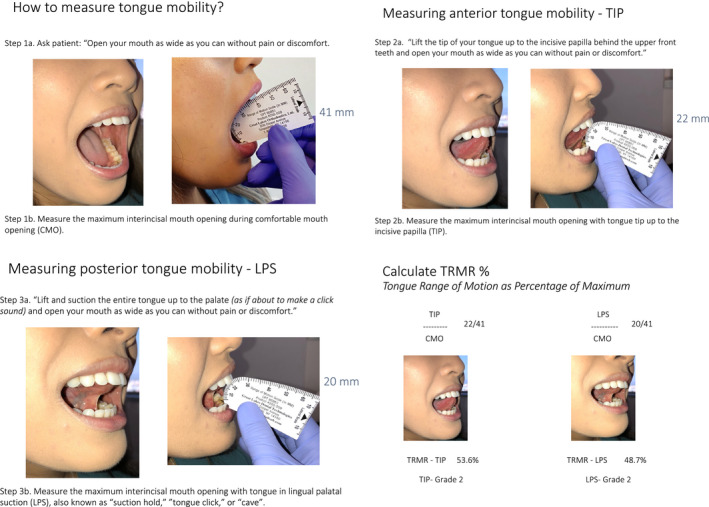
For assessment of anterior tongue mobility, maximum interincisal mouth opening with the tongue tip to the incisive papilla (TIP) is compared with the maximum interincisal mouth opening with the mouth opened as wide as possible without pain or discomfort (comfortable mouth opening, CMO); the percentage of TIP divided by CMO is defined as the TRMR‐TIP. For assessment of posterior tongue mobility, maximum interincisal mouth opening with the tongue in lingual‐palatal suction (LPS) is compared with CMO; the percentage of LPS divided by CMO is defined as the TRMR‐LPS. Note: Lingual‐palatal suction (LPS) is also described as ‘tongue suction’, ‘suction hold’, ‘tongue click and hold’ or ‘cave’ among the myofunctional therapy community

**FIGURE 2:**
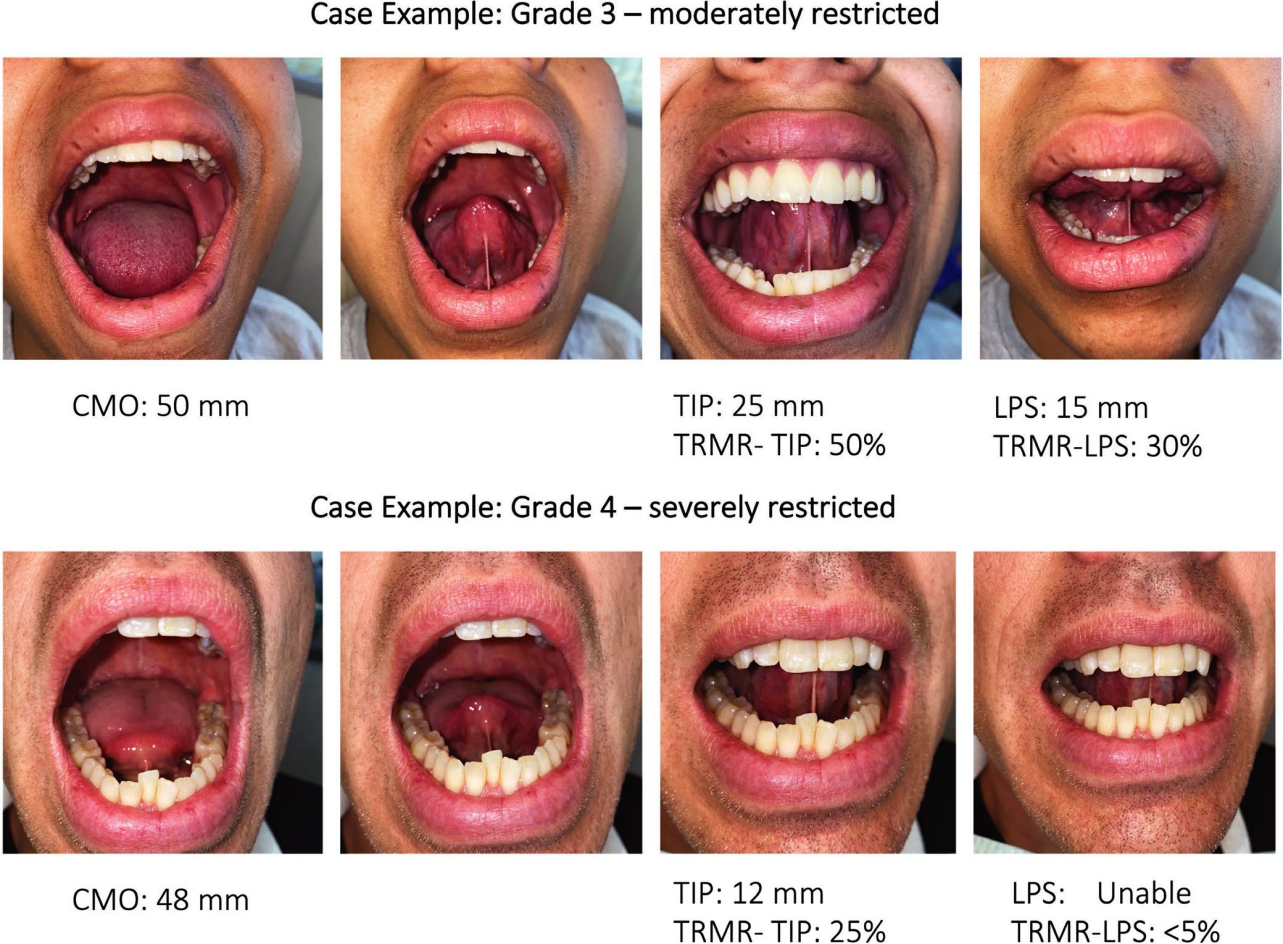
Case examples of moderately and severely restricted TIP and LPS tongue mobility

All measurements were obtained using a tongue range of motion instrument (Great Lakes Orthodontics) with the subjects sitting upright in a natural head position with a horizontal visual axis. For maximum interincisal mouth opening measurements, patients were instructed to open the mouth as wide as possible without pain or discomfort. The measurements were obtained on the first mouth opening to avoid jaw protrusion or excessive translation at the temporomandibular joint. For TIP measurements, subjects were instructed to ‘Lift the tip of your tongue up to the incisive papilla behind the upper front teeth and open your mouth as wide as you can without pain or discomfort’. This measurement is obtained with the tongue at the incisive papilla which is slightly anterior to ‘the spot’ landmark which is used during training with myofunctional therapy, see Figure 1. For LPS measurements, patients were instructed to ‘Lift and suction the entire tongue up to the palate (as if about to make a click sound) and open your mouth as wide as you can without pain or discomfort’, see Figure 2. Other objective assessments in this study included endurance of LPS (length of time that subjects could sustain lingual‐palatal suction) up to 30 seconds.

### 2.3 Subjective assessments

Other assessments included in the analysis for this manuscript from the FAIREST dataset included the following self‐assessment items rated subjectively on a 4‐point Likert scale: resting tongue position, ease or difficulty elevating the tongue tip to the palate, ease or difficulty elevating the tongue body to the palate; mouth breathing, slouching posture and positional sleep.

### 2.4 Clinical history

Measurements of mouth opening and tongue mobility were stratified based on the presence or absence of the following clinical history items: orthodontic treatment, myofunctional therapy, lingual frenectomy, tonsillectomy and temporomandibular joint disorder.

### 2.5 Statistical analysis

Statistical analyses were performed using JMP Pro 14 (SAS Institute Inc). Continuous variables are summarised as mean (M) ± standard deviation (SD) and standard error (SE) where applicable. Categorical variables are summarised as frequencies and percentages. Univariate analysis with Pearson's chi‐square or independent t test (continuous variables) was performed to assess for nominal or continuous covariates of TIP and LPS tongue mobility (mm IMO and % TRMR) vs. subjective reports of tongue mobility and clinical history. Due to the testing of multiple variables for each outcome, a two‐tailed *P*‐value < .01 was selected as the cut‐off for statistical significance.

## 3 RESULTS

**FIGURE 3:**
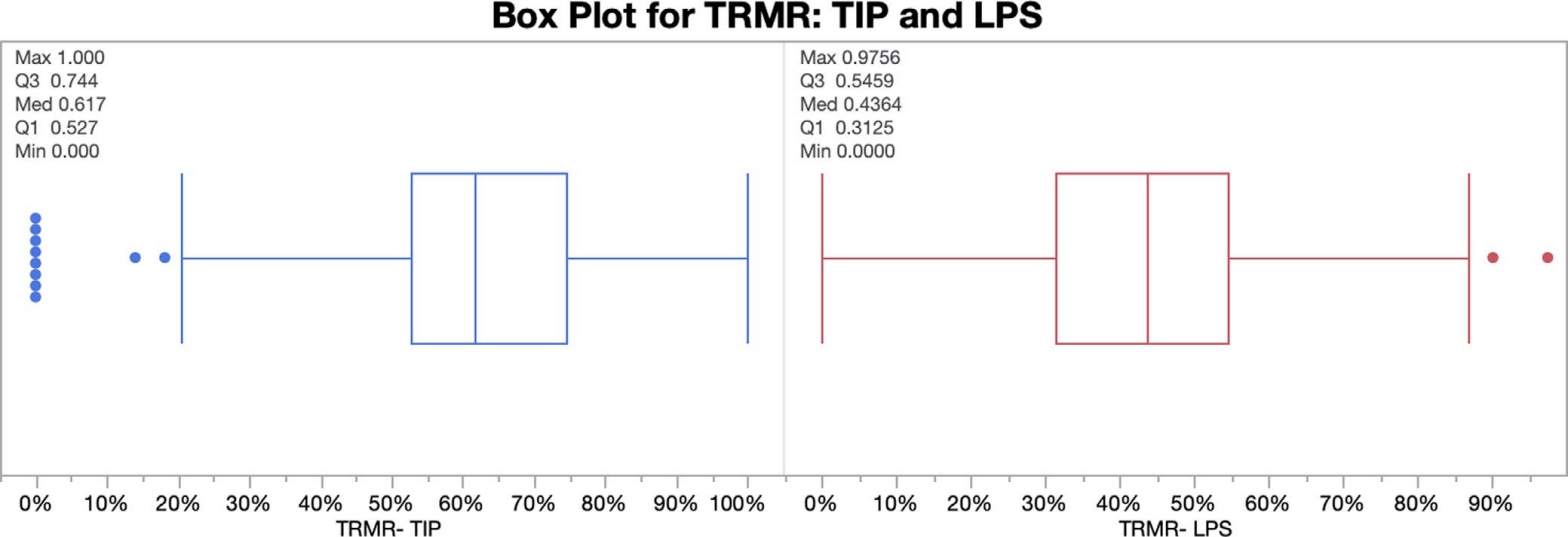
Distribution of measurements for TRMR‐TIP and TRMR‐LPS

**FIGURE 4:**
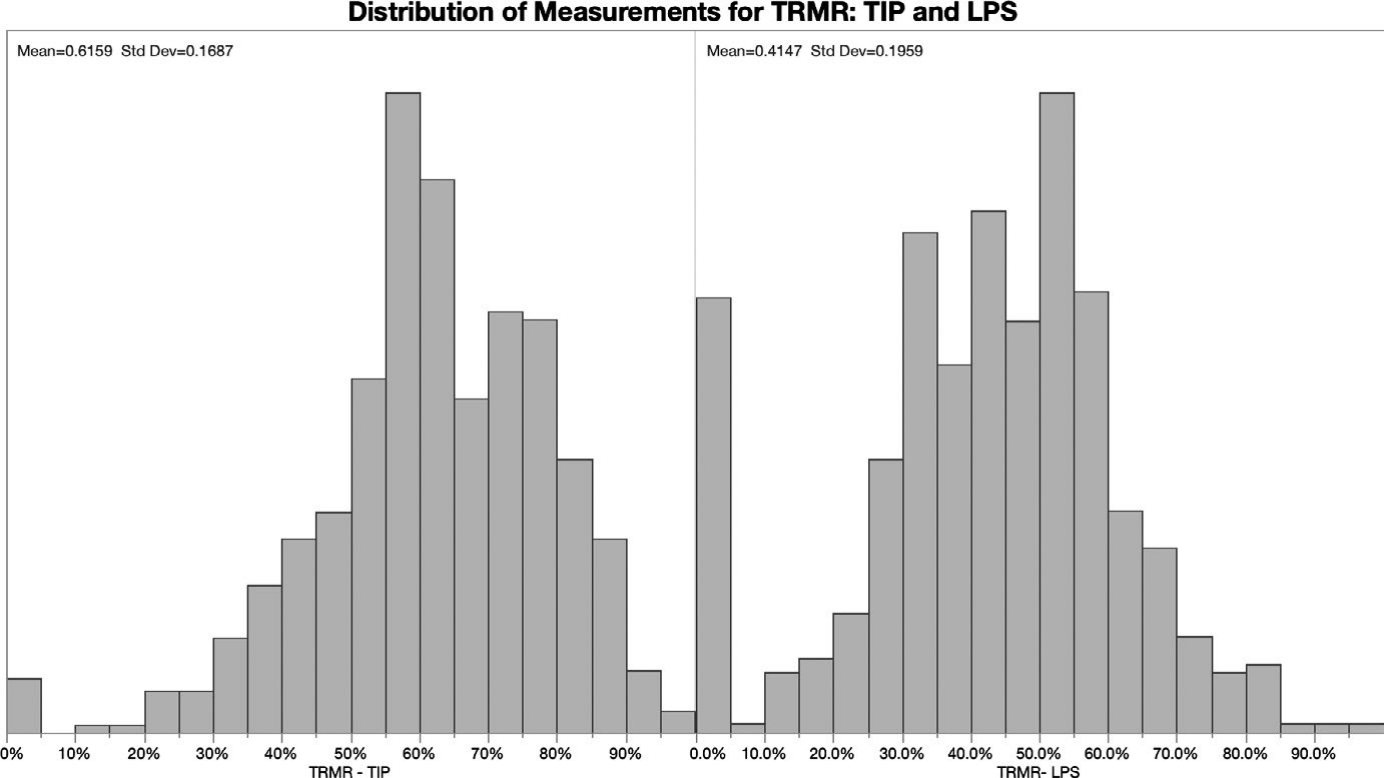
Box plot showing median and interquartile ranges for TRMR‐TIP and TRMR‐LPS

There were 611 subjects who participated in the tongue mobility assessments with average age: 20 ± 20 years (range 3‐83 years), including 23 pre‐school children (ages 3‐5), 257 grade‐school children (ages 3‐11), 75 adolescents (age 12‐17), 106 young adults (age 18‐35), 130 adults (age 36‐64) and 20 seniors (age ≥ 65). Gender distribution was 52.0% female. The overall mean ± standard deviation (SD) of the TRMR was 61.5 ± 16.8% for anterior tongue mobility (TIP) and 41.4 ± 19.5% for posterior tongue mobility (LPS), *P* < .0001, see Figures 3 and 4. Stratification by cohorts revealed that the tongue mobility and comfortable mouth opening measurements were modestly reduced in the child age cohort (*P* < .0001) but not significantly affected by gender (*P* = .1500), see Table 1.

**TABLE 1:** Comfortable mouth opening, tongue mobility and TRMR by age cohorts

	Subjects	CMO	TIP	LPS	TRMR‐TIP	TRMR‐LPS
Overall	611	43.7 ± 7.3	26.8 ± 8.1	18.6 ± 8.0	61.5 ± 16.8%	41.4 ± 19.5%
Age cohort						
Pre‐school children (3‐5 years)*	23	37.0 ± 6.3*	16.9 ± 9.1*	12.7 ± 9.0%*	47.1 ± 17.5%*	25.6% ± 26.4%*
Grade‐school children (5‐11 years)*	257	42.0 ± 6.3*	24.5 ± 7.4*	16.3 ± 7.7*	58.0 ± 16.3%*	36.7 ± 19.5%*
Adolescent (12‐17 years)	75	43.7 ± 6.8	29.9 ± 7.5	20.5 ± 7.0	61.0 ± 17.1%	45.4 ± 17.9%
Young adult (18‐35 years)	106	45.6 ± 7.5	30.8 ± 7.5	21.6 ± 6.6	67.7 ± 13.1%	46.1 ± 15.2%
Adult (36‐64 years)	130	45.1 ± 8.2	29.3 ± 7.5	21.1 ± 8.4	65.7 ± 13.1%	46.9 ± 18.6%
Senior (65 + years)	20	46.1 ± 8.3	30.2 ± 7.9	20.4 ± 8.8	66.8 ± 16.2%	46.5 ± 22.0%

Note

CMO, comfortable mouth opening; TIP, tongue to incisive papilla; LPS, lingual‐palatal suction; TRMR, tongue range of motion ratio.

* All measurements were significantly reduced in pre‐school and grade‐school age cohorts (ages 3‐11), *P* < .0001.

**FIGURE 5:**
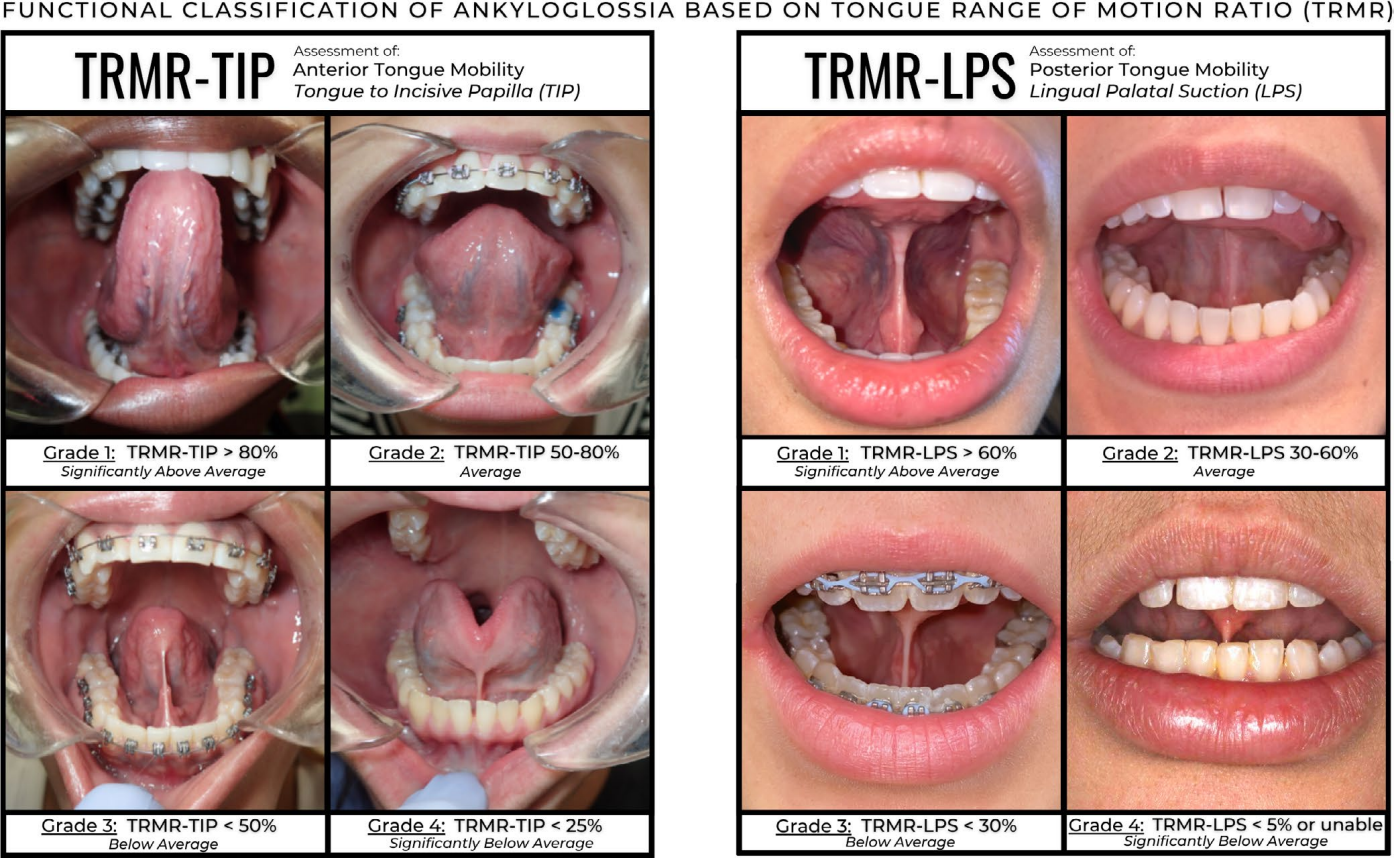
Updated grading scale for the functional classification of ankyloglossia based on the tongue range of motion ration (TRMR) performed with TIP and LPS—building on the previous classification proposed in Yoon et al 2017. Normative values and proposed grading scale are provided as TRMR‐TIP Grade 1 > 80%, Grade 2:50%‐80%, Grade 3: < 50%, Grade 4: < 25%; TRMR‐LPS Grade 1 > 60%, Grade 2:30%‐60%, Grade 3: <30%, Grade 4: <5% or unable to sustain. It should be noted that these measurements and grading scales may be unreliable in patients with limited mouth opening, strain and compensation patterns, children less than 12 years of age and any other patient who may not be able to follow the instructions for proper measurement

There was a statistically significant association between the objective measures of tongue mobility (TRMR‐TIP and TRMR‐LPS) and subjective reports of (a) difficulty elevating the tip of the tongue to the incisive papilla, (b) difficulty elevating the body of the tongue to the palate; and (c) tongue resting position. LPS measurements were much more highly correlated with differences in elevating the posterior body of the tongue as compared to TIP measurements (R^2^ 0.31 vs 0.05), see Figure 5.

Mean endurance for LPS was 21.1 ± 11.0 seconds. The endurance for LPS was significantly lower among patients with low resting tongue position (17.9 ± 12.2 seconds, *P* < .0001) as well patients with habitual mouth breathing (15.9 ± 12.6 seconds, *P* < .0001).

Patients with prior clinical history of myofunctional therapy, temporomandibular joint disorder and orthodontic treatment demonstrated mildly increased values for TRMR‐TIP and TRMR‐LPS. Other clinical history including whether the patient had a prior lingual frenectomy or tonsillectomy did not significantly impact TRMR measurements, see Table 2.

**TABLE 2:** Comfortable mouth opening, tongue mobility and TRMR by clinical history

	Subjects	CMO	TIP	LPS	TRMR‐TIP	TRMR‐LPS
Prior orthodontics						
No	427	42.9 ± 7.1	25.7 ± 8.1	17.6 ± 8.1	60.1 ± 17.4%	39.1 ± 20.4%
Yes	184	45.3 ± 7.3	29.3 ± 7.8	21.1 ± 7.4	64.9 ± 15.0%	46.9 ± 16.5%
		*P* = .0002	*P* < .0001	*P* < .0001	*P = *.0013	*P < *.0001
Prior myofunctional therapy						
No	579	43.7 ± 7.2	26.5 ± 8.0	18.2 ± 7.9	60.8 ± 16.6%	40.8 ± 19.5%
Yes	86	43.6 ± 7.7	28.8 ± 9.0	21.3 ± 8.2	66.1 ± 18.1%	45.7 ± 20.0%
		NS	*P* = .0146	*P* = .0011	*P* = .0066	*P* = .0291
Prior lingual frenectomy						
No	544	43.4 ± 7.3	26.6 ± 8.0	18.5 ± 7.9	61.6 ± 16.7%	41.3 ± 19.6%
Yes	67	45.9 ± 6.7	28.2 ± 9.4	20.2 ± 9.2	61.3 ± 18.3%	42.1 ± 19.9%
		*P* = .0082	NS	NS	NS	NS
Prior tonsillectomy						
No	608	43.5 ± 7.1	26.7 ± 8.2	18.5 ± 8.1	61.5 ± 17.0%	41.0 ± 19.8%
Yes	57	45.5 ± 8.5	28.1 ± 7.4	19.9 ± 7.6	62.8 ± 15.9%	45.0 ± 17.8%
		NS	NS	NS	NS	NS
Temporomandibular joint disorder						
No	578	43.7 ± 7.1	26.4 ± 8.3	18.2 ± 8.0	60.4 ± 16.9%	40.2 ± 19.4%
Yes	87	43.3 ± 8.2	29.4 ± 7.2	21.2 ± 7.7	68.8 ± 14.7%	49.8 ± 18.9%
		NS	*P* = .0020	*P* = .0019	*P* < .0001	*P* < .0001

## 4 DISCUSSION

The present study demonstrates normative values for anterior and posterior tongue mobility using TIP and LPS functional movements. The results in this study build on the work by Yoon et al^13^ and Marchesan.^25^ The prior studies helped establish and validate a functional approach to the assessment of ankyloglossia based on vertical extension of tongue mobility compared with mouth opening (described in this manuscript as the TRMR‐TIP assessment). TRMR‐TIP was found to be a more reliable tool for the functional assessment of tongue mobility in comparison with the traditional assessment of ankyloglossia which was based on the structural free‐tongue^17^ or frenulum length.^15^ Since that time, the TRMR‐TIP measurement has been used to demonstrate an association of restricted tongue mobility to development of the maxillary arch and elongation of the soft palate,^6^ as well as case selection in lingual frenuloplasty and myofunctional therapy for the treatment of mouth breathing, snoring, clenching and myofascial tension in appropriately selected patient candidates.^9^

In the present study, a cross‐sectional analysis was performed to take measurements of tongue mobility using TIP as well as LPS among subjects in the general population. This study validates TIP measurements as an effective assessment of anterior tongue mobility and LPS measurements as an effective assessment of posterior tongue mobility. The advantage of the LPS measurement is that it best describes one of the main functional outcome goals of myofunctional therapy: achieving tongue body to palate contact requisite for establishing ideal resting oral posture and swallow mechanics. LPS measurements have been used to track progress with tongue strengthening and rehabilitation in myofunctional, speech and swallow therapy protocols.^9^ Measurements for this study were taken at 10 sites internationally for maximal external validity, but it should be noted that the recruitment of friends and family of the researchers may have introduced selection bias that can affect generalisability.

The present work is one of the largest series in the literature with normative ranges and values for TIP and LPS in n = 611 subjects ages 3‐83 years, building on the prior report of frenulum length and TIP measurements in n = 200 children aged 6‐12 years by Ruffoli,^15^ n = 98 subjects with age > 18 years with TIP and LPS measurements by Marchesan^25^ and n = 1052 subjects ages six and up with TIP and Kotlow free‐tongue measurements by Yoon et al^13^ The present work identifies TIP‐TRMR of <50% and LPS‐TRMR of <30% to be considered as representative of moderately restricted anterior and posterior tongue mobility, respectively, among subjects ages 12‐65 + years. The results in this manuscript demonstrate that TRMR measurements may be unreliable in pre‐school and grade‐school children (ages 3‐11 years), see Table 3. In the prior work by Marchesan,^25^ LPS measurements were described, but abandoned because TIP measurements were found to be more highly associated with structurally apparent alterations of the lingual frenulum. We now appreciate the potential strength of the LPS measurements in identifying limitations in posterior tongue mobility that may be associated with functional deficits or submucosal restrictions that are not readily identified by other grading scales. It is important to emphasise that many factors can potentially impact functional movements of the tongue including but not limited to airway obstruction, lack of generalised practice, discoordination, maladaptive habits, tongue‐tie (ie restrictive lingual frenulum), intra‐oral fascia restrictions, extraoral fascia restrictions, neurogenic factors and tongue space limitations. Clinical factors should always be considered and tongue mobility measurements alone should not be used in isolation for treatment planning, especially in regard to decisions for frenulum surgery.

**TABLE 3:** Clinical grading scale for assessment of anterior and posterior tongue mobility using the tongue range of motion ratio (TRMR)

Grade	Description	Anterior tongue mobility	Posterior tongue mobility
		TRMR‐TIP	TRMR‐LPS
1	Highly above average^a^	>80%	>60%
2	Normal range^a^ (Mild to average)	50%‐80%	30%‐60%
3	Moderately restricted	<50%	<30%
4	Severely restricted	<25%	<5% or unable to do

^a^ Other clinical factors (including limited mouth opening, strain and compensation patterns) should be considered.

There are limitations to TIP and LPS because these assessments do not take into consideration compensation patterns that may affect the measurements. Common compensation mechanisms include floor of mouth elevation, neck engagement, jaw protrusion and facial grimace among others. Previously, we have shown that some patients augment the actual tongue elevation by engaging the neck muscles and elevating the floor of mouth as a possible compensation for restricted tongue mobility.^9^ Clinically, floor of mouth elevation can be controlled by providing the subject with feedback or by holding a gloved finger or grooved director behind the mandibular incisors and asking the patient to lift the tongue while pressing down on the floor of mouth. Assessment of resting tongue posture can also be assessed on CT scan^26^ or with BioGlo ophthalmic fluorescence dye applied to the tongue and UV light used to assess fluorescence in the palate. Each of these tools is limited as they only provide for an assessment in a single moment in time rather than usual or typical tongue resting position. Electropalatography with palatometer oral interfaces (such as SmartPalate System) could be used to allow subjects to assess tongue posture and oral movements in real time.^27^, ^28^, ^29^ Tongue pressure can be assessed with Iowa Oral Performance Instrument (IOPI) among other devices,^30^, ^31^ but are only reliable if performed in combination with electromyography of the neck and jaw to control the involvement of cervical and facial muscles that may confound intra‐oral tongue pressure measurements.^32^ The endurance in seconds with which the subjects can maintain lingual‐palatal suction may be a useful metric for investigation in future studies. Tables S1 and S2 are provided as potential resources for future research and clinical validation.

## 5 CONCLUSION

This study validates the TRMR in lingual‐palatal suction as a useful functional metric for assessment of posterior tongue mobility. We encourage future studies on the functional ankyloglossia to consider assessments of TRMR with tongue to incisive papilla for assessment of anterior tongue mobility and TRMR with tongue in lingual‐palatal suction for assessment of posterior tongue mobility. Normative values and proposed grading scale are provided as TRMR‐TIP Grade 1 > 80%, Grade 2:50%‐80%, Grade 3: <50%, Grade 4: <25%; TRMR‐LPS Grade 1 > 60%, Grade 2:30%‐60%, Grade 3: <30%, Grade 4: <5% or unable to sustain.

## CONFLICT OF INTEREST

None declared.

## AUTHOR CONTRIBUTIONS

SZ: Conception, design, data analysis, development of theory, manuscript preparation. SS: Project coordination, data coding, drafting of manuscript, administrative and technical support. CP: Conception, design, development of methods, project coordination, development of theory, manuscript revision, administrative and technical support. LC: Creation and development of theory, data interpretation, systematic review, editorial support, advice, guidance. SVP: Creation and development of theory, development of methods, acquisition of data, interpretation of results, editorial support, manuscript revision. ZP, DKN, TJ, BOC, KW, ML, JM and LM: Development of methods, acquisition of data, interpretation of results, editorial support. NA: Development of methods, acquisition of data, interpretation of results, manuscript revision. AY: Development of theory, data interpretation, systematic review, editorial support, advice, guidance.

## Supporting information

Table S1

Table S2

## Data Availability

The data that support the findings of this study are available from the corresponding author, Soroush Zaghi, upon reasonable request.
