# Supplementary material for: Assessment of posterior tongue mobility using lingual‐palatal suction: Progress towards a functional definition of ankyloglossia
Source: J Oral Rehabil. 2021 Jan 17;48(6):692–700. doi: 10.1111/joor.13144 (PMC8247966; doi:10.1111/joor.13144)
Supplement: Supplementary file 1 — Table S1 [file JOOR-48-692-s001.docx]

**Supplemental Data Tables**

**Table S1.** Sub-classification of the proposed grading scale for assessment of anterior and posterior tongue mobility using the tongue range of motion ratio (TRMR). The additional 2a-2d categories are provided as a potential resource for future research and clinical validation.


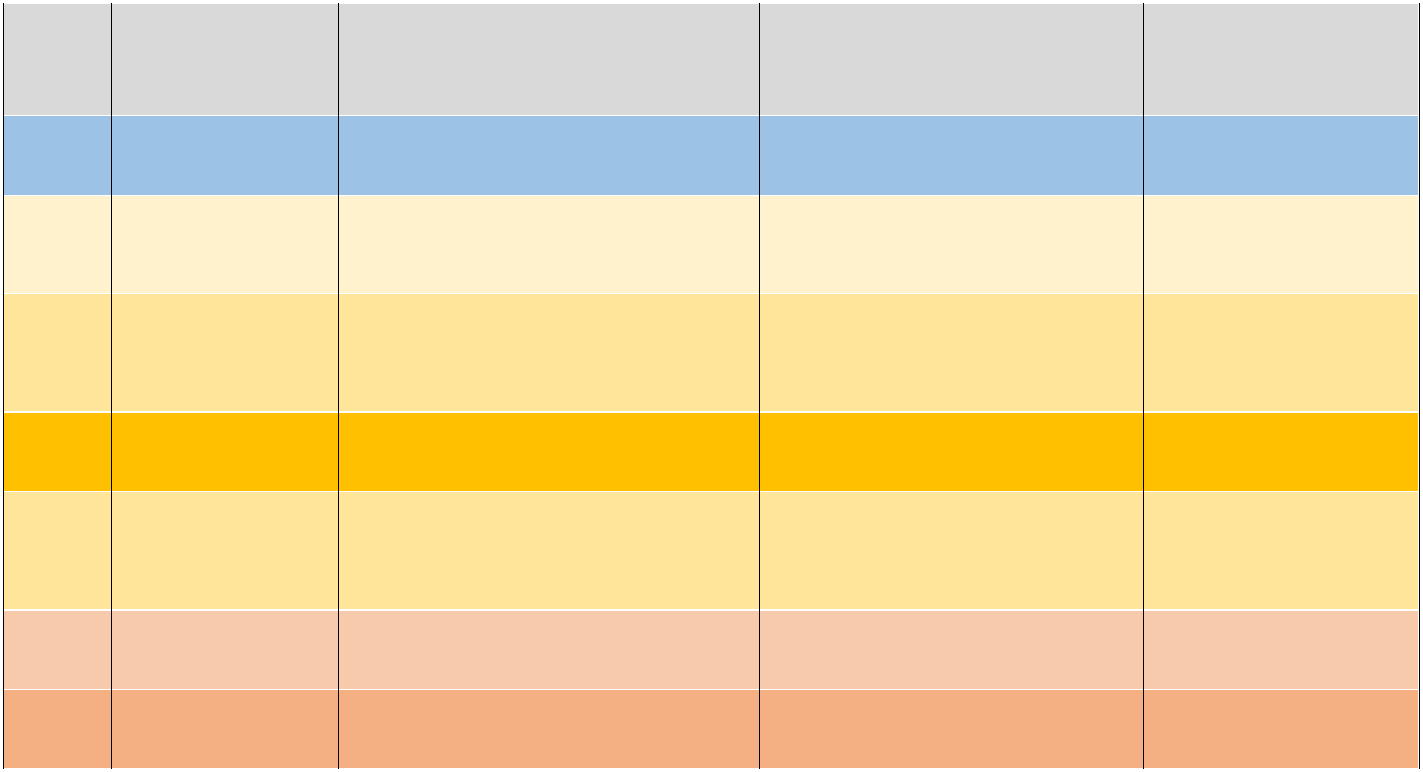


|  | Grade |  |  |  | Description | |  | Anterior Tongue Mobility: | | |  | Posterior Tongue Mobility: | | |  | Distribution | | |
| --- | --- | --- | --- | --- | --- | --- | --- | --- | --- | --- | --- | --- | --- | --- | --- | --- | --- | --- |
|  |  |  |  |  |  |  |  |  | **TRMR-TIP** |  |  |  | **TRMR LPS** |  |  |  |  |  |
|  | |  |  |  | | |  |  | |  |  |  | |  |  |  | | |
| 1 | |  |  | Significantly | | |  | >80% | |  |  | >60% | |  |  | Highest Decile | | |
|  |  |  |  | Above Average | | |  |  |  |  |  |  |  |  |  | (>90^th^ Percentile) | | |
| 2a | |  |  | Above Average | | |  | 70-80% | |  |  | 50-60% | |  |  | Upper Quartile | | |
|  |  |  |  |  |  |  |  |  |  |  |  |  |  |  |  | (>75^th^ Percentile) | | |
| 2b | |  |  | Slightly Above | | |  | 65-70% | |  |  | 45-50% | |  |  | Upper | | |
|  |  |  |  | Average | | |  |  |  |  |  |  |  |  |  | Interquartile | | |
|  |  |  |  |  |  |  |  |  |  |  |  |  |  |  |  | Range | | |
| 2c | |  |  | Average | | |  | 60±5% | |  |  | 40±5% | |  |  | Median (50^th^ | | |
|  |  |  |  |  |  |  |  |  |  |  |  |  |  |  |  | Percentile) | | |
| 2d | |  |  | Slightly Below | | |  | 50-55% | |  |  | 30-35% | |  |  | Lower | | |
|  |  |  |  | Average | | |  |  |  |  |  |  |  |  |  | Interquartile | | |
|  |  |  |  |  |  |  |  |  |  |  |  |  |  |  |  | Range | | |
| 3 | |  |  | Below Average | | |  | 25-50% | |  |  | 5-30% | |  |  | Lower Quartile | | |
|  |  |  |  |  |  |  |  |  |  |  |  |  |  |  |  | (<25^th^ Percentile) | | |
| 4 | |  |  | Significantly | | |  | <25% | |  |  | <5% | |  |  | Lowest Decile | | |
|  |  |  |  | Below Average | | |  |  |  |  |  |  | or unable | |  | (<10^th^ Percentile) | | |
