# Supplementary material for: Assessment of posterior tongue mobility using lingual‐palatal suction: Progress towards a functional definition of ankyloglossia
Source: J Oral Rehabil. 2021 Jan 17;48(6):692–700. doi: 10.1111/joor.13144 (PMC8247966; doi:10.1111/joor.13144)
Supplement: Supplementary file 2 — Table S2 [file JOOR-48-692-s002.docx]

**Table S2.** Normative values of the maximum interincisal mouth opening (IMO) during each of the three functional conditions from the distribution in this study population: comfortable mouth opening (CMO), tongue to incisive papilla (TIP), Lingual Palatal Suction (LPS). This reference table may be a potential resource for future research and clinical validation.


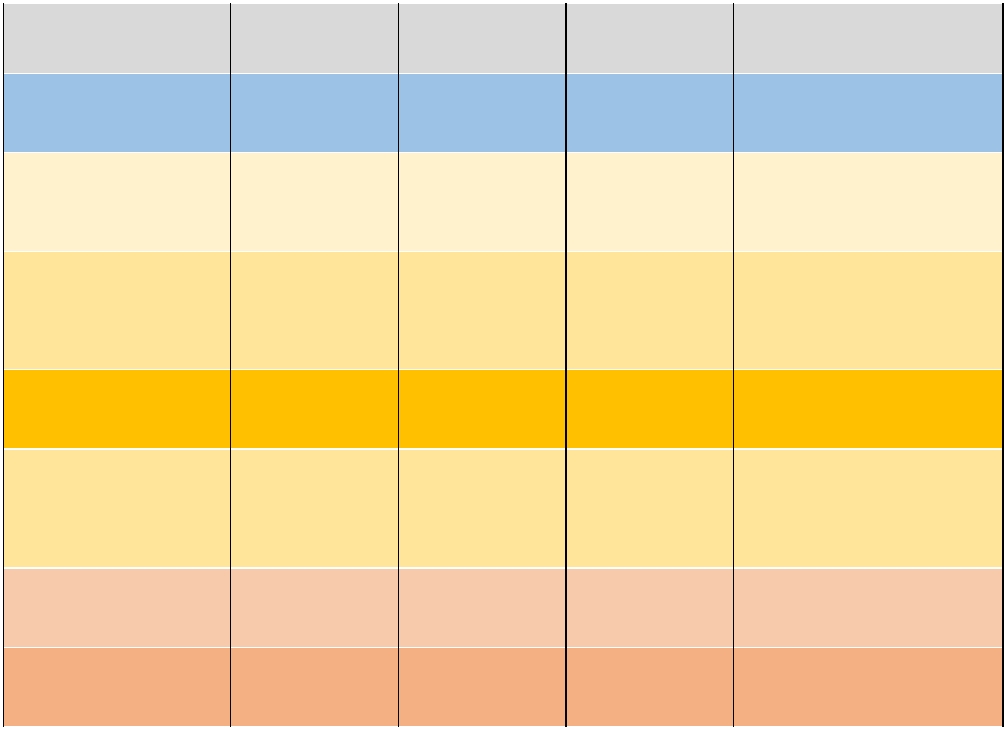


| Description | | |  | **CMO** |  | **TIP** | **LPS** | **Distribution** | | |
| --- | --- | --- | --- | --- | --- | --- | --- | --- | --- | --- |
|  |  |  |  | (IMO, mm) |  | (IMO, mm) | (IMO, mm) |  |  |  |
| Significantly | | |  | >52 mm |  | >34 mm | >28 mm | Highest Decile | | |
| Above Average | | |  |  |  |  |  | (>90^th^ Percentile) | | |
| Above Average | | |  | 50 mm |  | 32 mm | 26 mm | Upper Quartile | | |
|  |  |  |  |  |  |  |  | (>75^th^ Percentile) | | |
| Slightly Above | | |  | 46 mm |  | 28 mm | 22 mm | Upper | | |
| Average | | |  |  |  |  |  | Interquartile | | |
|  |  |  |  |  |  |  |  | Range | | |
| Average | | |  | 44 ± 2 mm |  | 26 ± 2 mm | 20 ± 2 mm | Median (50^th^ | | |
|  |  |  |  |  |  |  |  | Percentile) | | |
| Slightly Below | | |  | 42 mm |  | 24 mm | 18 mm | Lower | | |
| Average | | |  |  |  |  |  | Interquartile | | |
|  |  |  |  |  |  |  |  | Range | | |
| Below Average | | |  | 40 mm |  | 22 mm | 16 mm | Lower Quartile | | |
|  |  |  |  |  |  |  |  | (<25^th^ Percentile) | | |
| Significantly | | |  | <34 mm |  | <16 mm | <10 mm | Lowest Decile | | |
| Below Average | | |  |  |  |  | or unable | (<10^th^ Percentile) | | |
